# Kidney Stone Disease: An Update on Current Concepts

**DOI:** 10.1155/2018/3068365

**Published:** 2018-02-04

**Authors:** Tilahun Alelign, Beyene Petros

**Affiliations:** ^1^Department of Microbial, Cellular and Molecular Biology, College of Natural Sciences, Addis Ababa University, P.O. Box 1176, Addis Ababa, Ethiopia; ^2^Department of Biology, Debre Birhan University, P.O. Box 445, Debre Birhan, Ethiopia

## Abstract

Kidney stone disease is a crystal concretion formed usually within the kidneys. It is an increasing urological disorder of human health, affecting about 12% of the world population. It has been associated with an increased risk of end-stage renal failure. The etiology of kidney stone is multifactorial. The most common type of kidney stone is calcium oxalate formed at Randall's plaque on the renal papillary surfaces. The mechanism of stone formation is a complex process which results from several physicochemical events including supersaturation, nucleation, growth, aggregation, and retention of urinary stone constituents within tubular cells. These steps are modulated by an imbalance between factors that promote or inhibit urinary crystallization. It is also noted that cellular injury promotes retention of particles on renal papillary surfaces. The exposure of renal epithelial cells to oxalate causes a signaling cascade which leads to apoptosis by p38 mitogen-activated protein kinase pathways. Currently, there is no satisfactory drug to cure and/or prevent kidney stone recurrences. Thus, further understanding of the pathophysiology of kidney stone formation is a research area to manage urolithiasis using new drugs. Therefore, this review has intended to provide a compiled up-to-date information on kidney stone etiology, pathogenesis, and prevention approaches.

## 1. Introduction

### 1.1. Overview of Kidney Stones

Kidney stones are mainly lodged in the kidney(s) [1]. Mankind has been afflicted by urinary stones since centuries dating back to 4000 B.C. [2], and it is the most common disease of the urinary tract. The prevention of renal stone recurrence remains to be a serious problem in human health [3]. The prevention of stone recurrence requires better understanding of the mechanisms involved in stone formation [4]. Kidney stones have been associated with an increased risk of chronic kidney diseases [5], end-stage renal failure [3, 6], cardiovascular diseases [7, 8], diabetes, and hypertension [9]. It has been suggested that kidney stone may be a systemic disorder linked to the metabolic syndrome. Nephrolithiasis is responsible for 2 to 3% of end-stage renal cases if it is associated with nephrocalcinosis [10].

The symptoms of kidney stone are related to their location whether it is in the kidney, ureter, or urinary bladder [11]. Initially, stone formation does not cause any symptom. Later, signs and symptoms of the stone disease consist of renal colic (intense cramping pain), flank pain (pain in the back side), hematuria (bloody urine), obstructive uropathy (urinary tract disease), urinary tract infections, blockage of urine flow, and hydronephrosis (dilation of the kidney). These conditions may result in nausea and vomiting with associated suffering from the stone event [12]. Thus, the treatment and time lost from work involves substantial cost imposing an impact on the quality of life and nation's economy.

### 1.2. Epidemiology of Kidney Stones

Globally, kidney stone disease prevalence and recurrence rates are increasing [13], with limited options of effective drugs. Urolithiasis affects about 12% of the world population at some stage in their lifetime [14]. It affects all ages, sexes, and races [15, 16] but occurs more frequently in men than in women within the age of 20–49 years [17]. If patients do not apply metaphylaxis, the relapsing rate of secondary stone formations is estimated to be 10–23% per year, 50% in 5–10 years, and 75% in 20 years of the patient [15]. However, lifetime recurrence rate is higher in males, although the incidence of nephrolithiasis is growing among females [18]. Therefore, prophylactic management is of great importance to manage urolithiasis.

Recent studies have reported that the prevalence of urolithiasis has been increasing in the past decades in both developed and developing countries. This growing trend is believed to be associated with changes in lifestyle modifications such as lack of physical activity and dietary habits [19–21] and global warming [16]. In the United States, kidney stone affects 1 in 11 people [22], and it is estimated that 600,000 Americans suffer from urinary stones every year. In Indian population, about 12% of them are expected to have urinary stones and out of which 50% may end up with loss of kidney functions [23].

## 2. The Urinary System and Stones

**Figure 1:**
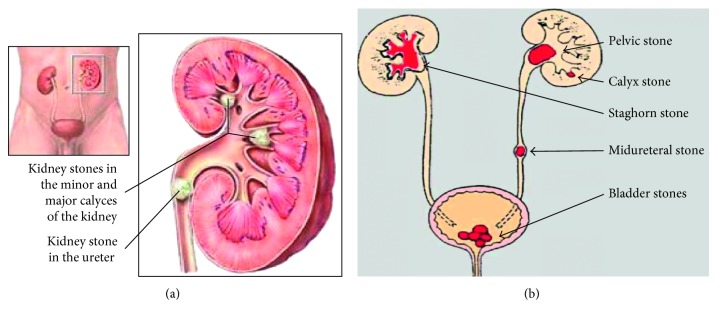
Kidney stone locations in the urinary system. (a) Adopted from [25]. (b) Adopted from [26].

The urinary filtrate is formed in the glomerulus and passes into the tubules where the volume and content are altered by reabsorption or secretions. Most solute reabsorption occurs in the proximal tubules, whereas fine adjustments to urine composition take place in the distal tubule and collecting ducts. The loop of Henle serves to concentrate urine composed of 95% water, 2.5% urea, 2.5% mixture of minerals, salts, hormones, and enzymes. In the proximal tubules, glucose, sodium, chloride, and water are reabsorbed and returned to the blood stream along with essential nutrients such as amino acids, proteins, bicarbonate, calcium, phosphate, and potassium. In the distal tubule, the salt and acid-base balance of blood is regulated [24]. The location of stones may vary as indicated in Figure 1.

## 3. Types of Kidney Stones

The chemical composition of kidney stones depends on the abnormalities in urine composition of various chemicals. Stones differ in size, shape, and chemical compositions (mineralogy) [27]. Based on variations in mineral composition and pathogenesis, kidney stones are commonly classified into five types as follows [28].

### 3.1. Calcium Stones: Calcium Oxalate and Calcium Phosphate

Calcium stones are predominant renal stones comprising about 80% of all urinary calculi [29]. The proportion of calcium stones may account for pure calcium oxalate (CaOx) (50%), calcium phosphate (CaP, termed as apatite) (5%), and a mixture of both (45%) [30]. The main constituent of calcium stones is brushite (calcium hydrogen phosphate) or hydroxyapatite [31, 32]. Calcium oxalate is found in the majority of kidney stones and exists in the form of CaOx monohydrate (COM, termed as mineral names: whewellite, CaC_2_O_4_·H_2_O), and CaOx dihydrate (COD, weddellite, CaC_2_O_4_·2H_2_O), or as a combination of both which accounts for greater than 60% [33]. COM is the most thermodynamically stable form of stone. COM is more frequently observed than COD in clinical stones [34].

Many factors contribute to CaOx stone formation such as hypercalciuria (resorptive, renal leak, absorptive, and metabolic diseases), hyperuricosuria, hyperoxaluria, hypocitraturia, hypomagnesuria, and hypercystinuria [35]. Mostly, urinary pH of 5.0 to 6.5 promotes CaOx stones [36], whereas calcium phosphate stones occur when pH is greater than 7.5 [11]. The recurrence of calcium stone is greater than other types of kidney stones.

### 3.2. Struvite or Magnesium Ammonium Phosphate Stones

Struvite stones occur to the extent of 10–15% and have also been referred to as infection stones and triple phosphate stones. It occurs among patients with chronic urinary tract infections that produce urease, the most common being *Proteus mirabilis* and less common pathogens include *Klebsiella pneumonia*, *Pseudomonas aeruginosa,* and *Enterobacter* [1, 28, 29]. Urease is necessary to split/cleave urea to ammonia and CO_2,_ making urine more alkaline which elevates pH (typically > 7). Phosphate is less soluble at alkaline versus acidic pH, so phosphate precipitates on to the insoluble ammonium products, yielding to a large staghorn stone formation [37]. Women's are likely to develop this type of stone than the male. *Escherichia coli* is not capable of splitting urea and is not associated with struvite stones [38].

### 3.3. Uric Acid Stones or Urate

This accounts approximately for 3–10% of all stone types [1, 29]. Diets high in purines especially those containing animal protein diet such as meat and fish, results in hyperuricosuria, low urine volume, and low urinary pH (pH < 5.05) exacerbates uric acid stone formation [11, 28, 39]. Peoples with gouty arthritis may form stones in the kidney(s). The most prevalent cause of uric acid nephrolithiasis is idiopathic [38], and uric acid stones are more common in men than in women.

### 3.4. Cystine Stones

These stones comprise less than 2% of all stone types. It is a genetic disorder of the transport of an amino acid and cystine. It results in an excess of cystinuria in urinary excretions [1, 29], which is an autosomal recessive disorder caused by a defect in the rBAT gene on chromosome 2 [40], resulting in impaired renal tubular absorption of cystine or leaking cystine into urine. It does not dissolve in urine and leads to cystine stone formation [11]. People who are homozygous for cystinuria excrete more than 600 millimole insoluble cystine per day [28]. The development of urinary cystine is the only clinical manifestation of this cystine stone disease [40].

### 3.5. Drug-Induced Stones

This accounts for about 1% of all stone types [1]. Drugs such as guaifenesin, triamterene, atazanavir, and sulfa drugs induce these stones. For instance, people who take the protease inhibitor indinavir sulphate, a drug used to treat HIV infection, are at risk of developing kidney stones [28]. Such lithogenic drugs or its metabolites may deposit to form a nidus or on renal calculi already present. On the other hand, these drugs may induce the formation of calculi through its metabolic action by interfering with calcium oxalate or purine metabolisms [38].

## 4. Kidney Stone Compositions

The chemical compositions of urinary stones include crystals and noncrystalline phases or the organic material (the matrix). The organic matrix of urinary stones consists of macromolecules such as glycosaminoglycans (GAG's), lipids, carbohydrates, and proteins. These molecules play a significant role by promoting or inhibiting the processes of kidney stone development (Table 1). The main components of the stone matrix are proteins (64%), nonamino sugars (9.6%), hexosamine as glucosamine (5%), water (10%), and inorganic ash (10.4%). The matrix acts as a template participating in the assembly of kidney stones. The matrix of all stones contains phospholipids (8.6%) of the total lipid, which in turn represents about 10.3% of stone matrix. Cell membrane phospholipids, as part of organic matrix, promote the formation of calcium oxalate and calcium phosphate stones [41]. Albumin is the major component of the matrix of all stone types [42].

Brushite stone is a hard phosphate mineral with an increasing incidence rate, and a quarter of calcium phosphate (CaP) patients form stones containing brushite [43]. In the urinary tract, CaP may be present in the form of hydroxyapatite, carbonate apatite, or brushite (calcium monohydrogen phosphate dihydrate, CaHPO4·2H2O). Brushite is resistant to shock wave and ultrasonic lithotripsy treatment [44].

### 4.1. Etiology of Kidney Stones

Formation of kidney stones (calculogenesis) is a complex and multifactorial process including intrinsic (such as age, sex, and heredity) and extrinsic factors such as geography, climate, dietary, mineral composition, and water intake [15]. A summary of possible causes of kidney stone formation is shown in Table 2.

## 5. Mechanisms of Renal Stone Formation

The pathogenesis of kidney stone or biomineralization is a complex biochemical process which remains incompletely understood [41]. Renal stone formation is a biological process that involves physicochemical changes and supersaturation of urine. Supersaturated solution refers to a solution that contains more of dissolved material than could be dissolved by the solvent under normal circumstances [34]. As a result of supersaturation, solutes precipitate in urine leads to nucleation and then crystal concretions are formed. That is, crystallization occurs when the concentration of two ions exceeds their saturation point in the solution [55]. The transformation of a liquid to a solid phase is influenced by pH and specific concentrations of excess substances. The level of urinary saturation with respect to the stone-forming constituents like calcium, phosphorus, uric acid, oxalate, cystine, and low urine volume are risk factors for crystallization [1, 56]. Thus, crystallization process depends on the thermodynamics (that leads to nucleation) and kinetics (which comprises the rates of nucleation or crystal growth) of a supersaturated solution [57]. Therefore, lithiasis can be prevented by avoiding supersaturation.

However, it should be noted that stone formation is usually dependent on the level of imbalance between urinary inhibitors and promoters of crystallization. All stones share similar events with respect to the mineral phase of stone formation. But, the sequence of events leading to stone formation differs depending on the type of stone and urine chemistry. For instance, crystallization of calcium-based stones (calcium oxalate or calcium phosphate) occurs in supersaturated urine if it is with low concentrations of inhibitors. Uric acid interferes the solubility of calcium oxalate and promotes CaOx stone formation. In healthy controls, crystallization process is opposed by inhibitory substances and gets safe [1]. The sequence of events that trigger stone formation includes nucleation, growth, aggregation, and retention of crystals within the kidneys [27, 58].

### 5.1. Crystal Nucleation

The first step in the formation of kidney stone begins by the formation of nucleus (termed as nidus) from supersaturated urine retained inside the kidneys [11, 42]. In a supersaturated liquid, free atoms, ions, or molecules start forming microscopic clusters that precipitate when the bulk free energy of the cluster is less than that of the liquid. For example, charged soluble molecules such as calcium and oxalate combine to form calcium oxalate crystals and become insoluble [34]. Nucleation may be formed in the kidney through free particle or fixed particle mechanism [26, 34]. In supersaturated solutions, if promoters exceed that of inhibitors, nucleation starts [34].

Once a nucleus is created (and/or if it is anchored), crystallization can occur at lower chemical pressure than required for the formation of the initial nucleus. Existing epithelial cells, urinary casts, RBCs, and other crystals in urine can act as nucleating centers in the process of nuclei formation termed as heterogeneous nucleation [41]. The organic matrix, mucopolysaccharide acts as a binding agent by increasing heterogeneous nucleation and crystal aggregation [59]. On the other hand, nanobacteria is claimed to form apatite structures serving as a crystallization center for stone formation [60]. The whole process potentiates stone formation. The role of oxalate-degrading bacteria, such as *Oxalobacter formigenes*, in CaOx stone formation is a subject of current research [61]. Thus, treatment which targets the process of nucleation intervention is one of the best approaches to control kidney stone.

### 5.2. Crystal Growth

Crystals in urine stick together to form a small hard mass of stone referred as crystal growth. Stone growth is accomplished through aggregation of preformed crystals or secondary nucleation of crystal on the matrix-coated surface [62]. Once a nidus has achieved, the overall free energy is decreased by adding new crystal components to its surface. The total free energy of the cluster is increased by the surface energy. The process of stone growth is slow and requires longer time to obstruct the renal tubules [34]. From organic matrix, mainly Tamm–Horsfall protein and osteopontin are promoters of CaOx stone formation [13]. Under in vitro study, crystals induced in human urine demonstrated an intimate association between calcium-containing crystals and organic matrix (lipids and proteins). Lipids of cellular membranes are basically believed to involve in nucleation of crystals [63].

### 5.3. Crystal Aggregation

The process whereby a small hard mass of a crystal in solution sticks together to form a larger stone is called aggregation. All models of CaOx urolithiasis concede that crystal aggregation is probably involved in crystal retention within the kidneys [41]. Crystal aggregation is considered to be the most critical step in stone formation.

### 5.4. Crystal-Cell Interaction

The attachment of grown crystals with the renal tubule lining of epithelial cells is termed as crystal retention or crystal-cell interaction [41, 64]. In individuals with hyperoxaluria, renal tubular epithelial cells were injured due to exposure to high oxalate concentrations or sharp calcium oxalate monohydrate (COM) crystals [10, 65, 66]. Crystal-cell interaction results in the movement of crystals from basolateral side of cells to the basement membrane [10]. Then, crystals could be taken into cells and anchored to the basement membrane of the kidneys [66]. The interaction of COM crystals with the surface of renal epithelial cells could be a critical initiating event in nephrolithiasis. An increased retention force between the crystal and injured renal tubule epithelium cells promotes CaOx crystallization [67]. Most of the crystals attached to epithelial cells are thought to be digested by macrophages and/or lysosomes inside cells and then discharged with urine [66].

Following renal tubular cell injury, cellular degradation produces numerous membrane vesicles which are nucleators of calcium crystals as supported by in vitro and in vivo studies [41]. Injured cells release substances like renal prothrombin fragment-1 or other anionic proteins which induce COM crystal agglomeration [68]. Reactive oxygen species is thought to be one of the factors involved in renal cell injury [69]. Thus, reduction of renal oxidative stress could be an effective treatment option.

Injured cells potentiate to invert its cell membrane which is anionic to the urinary environment and acts as site of crystal adherence. COM crystals have stronger affinity of attachment towards the inverted anionic membrane [69], than calcium oxalate dihydrate (COD) crystals [70]. On the other hand, deposition of COM crystal was observed in Madin–Darby canine kidney epithelial cells (MDCK cells), than at proximal tubular epithelial cells derived from pig kidney (LLC-PK1 cells) study models [71]. This preferential difference may be due to the presence of a binding molecule such as hyaluronan on Madin–Darby canine kidney epithelial cells for COM crystal attachment [67]. Although the detailed mechanisms of crystal-cell interaction remain unexplored, one of the best ways to treat urolithiasis is to control crystal-cell retentions.

### 5.5. Endocytosis of CaOx Crystals

Endocytosis or engulfment of crystals by renal tubular cells is the earliest process in the formation of kidney stones. Studies on tissue culture crystal-cell interactions indicated that COM crystals rapidly adhere to microvilli on the cell surface and subsequently internalized. Polyanion molecules present in tubular fluid/urine such as glycosaminoglycans, glycoproteins, and citrate may coat crystals and inhibit the binding of COM crystals to cell membrane [41]. For example, Tamm–Horsfall glycoproteins (THP) have a dual biological role in stone formation. Lieske et al. [72] reported that THP may promote renal stone formation by initiating the interaction of COM crystals with distal tubular cells of the nephron. Another study revealed that, upon lowering pH and raising ionic strength, THP's viscosity increases which exhibits high tendency of polymerization and fails to inhibit crystallization. Moreover, THP becomes a strong promoter of crystallization in the presence of additional calcium ions [73]. In contrast, THP is thought to protect against COM stone formation by inhibiting COM aggregation when it is at high pH and low ionic strength as reported by Hess [73]. COM aggregation assays revealed that desialylated THP promoted COM aggregation, while normal THP inhibited aggregation [74]. Similar reports revealed that THP may inhibit calcium oxalate crystal aggregation, whereas uromodulin may promote aggregation [75]. Inactivating the THP gene in mouse embryonic stem cells results in spontaneous formation of calcium crystals in adult kidneys. This is a convincing evidence that THP is a critical urinary inhibitor of human nephrolithiasis [76].

**Figure 2:**
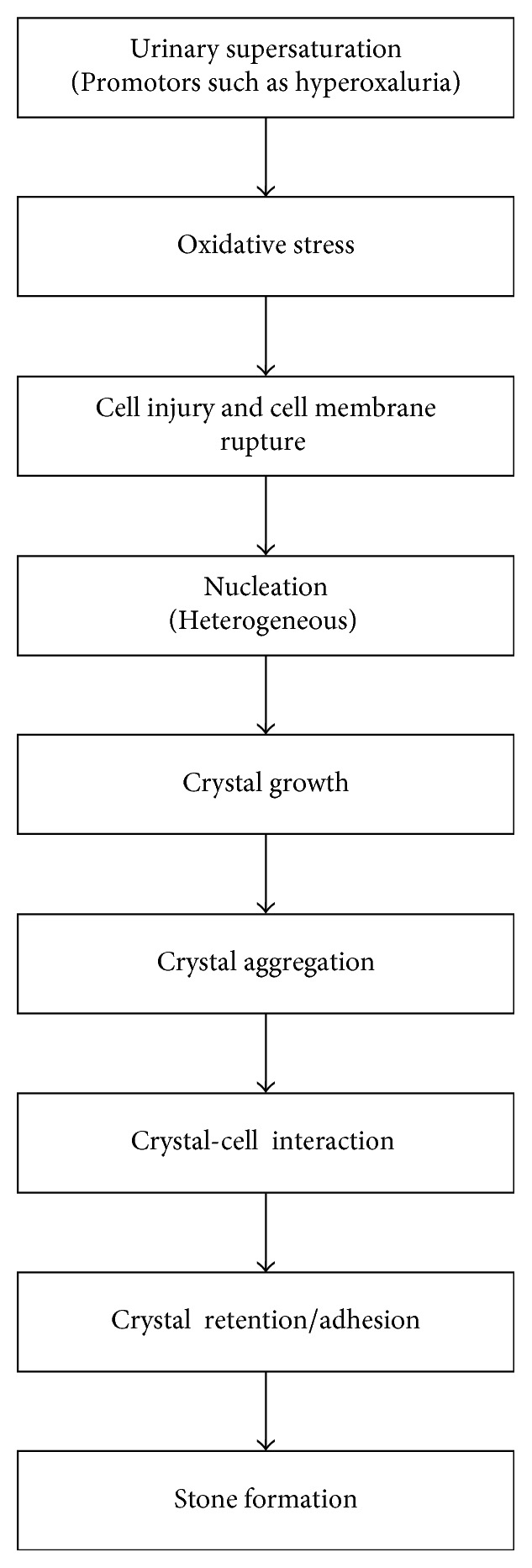
Schematic representation of the various events of kidney stone formation.

Various cellular and extracellular events are involved during stone formation. Modulators targeting the steps from supersaturation to crystal retention may be a potential means to block stone formation. Similarly, the blockage of crystal binding molecules (such as osteopontin, hyaluronic acid, sialic acid, and monocyte chemoattractant protein-1) expressed on epithelial cell membranes may be an alternative approach to prevent stone formation [41]. Experimental findings demonstrated that stone calcification is triggered by reactive oxygen species (ROS) and the development of oxidative stress [77]. In vitro [78, 79] and in vivo [80, 81] studies have demonstrated that CaOx crystals are toxic for renal epithelial cells that produce injury and renal cell death. Similarly, an exposure to hypercalciuria produces cellular injury and ROS-induced lipid peroxidation which stimulates calcium oxalate deposition [82]. The pathophysiology of urinary stone formation is incompletely understood. A summary of the various steps involved in stone formation is shown below (Figure 2).

### 5.6. Cell Injury and Apoptosis

Exposure to high levels of oxalate or CaOx crystals induces epithelial cellular injury, which is a predisposing factor to subsequent stone formation [83, 84]. CaOx crystal depositions in the kidneys upregulate the expression and synthesis of macromolecules that can promote inflammation [85]. Crystals may be endocytosed by cells or transported to the interstitium. It has been suggested that injured cells develop a nidus which promotes the retention of particles on the renal papillary surface [86]. In individuals with severe primary hyperoxaluria, renal tubular cells are injured and crystals become attached to them [66]. The addition of CaOx crystals onto Madin–Darby canine kidney (MDCK) cell lines showed an increase in the release of lysosomal enzymes, prostaglandin E2, and cytosolic enzymes [87]. A study on animal models also revealed that the administration of high concentrations of CaOx crystals or oxalate ions appears to be toxic causing renal tubular cell damage [41]. It has been suggested that oxalate increases the availability of free radicals by inhibiting enzymes responsible for their degradation. For instance, reactive oxygen species can damage the mitochondrial membrane and reduce its transmembrane potential. These events are known features of early process in apoptotic pathways [88].

The activation of p38 mitogen-activated protein kinase (p38 MAPK) signaling pathway regulates the expression of cellular proteins. The various extracellular stimuli or stresses like ultraviolet radiation and proinflammatory cytokines may activate p38 MAPK which results in phosphorylation and activation of transcription factors [89]. The exposure of renal cells to oxalate increases an altered gene expression that induces apoptosis signaling cascades [88]. A study revealed that the exposure of HK-2 cells to increased oxalate levels results in an increased transcriptional activation of IL-2R beta mRNA and consequently increases IL-2R beta protein levels which drive cellular changes like induction of inflammation. Oxalate-induced activation may trigger p38 MAPK signaling by acting on cell membranes, although the exact mechanisms have not been established [90].

Apoptosis at the level of renal tubular cells may lead to stone formation through cellular demise and postapoptotic necrosis which could promote calcium crystal aggregation and growth. This fact has been supported by in vitro study on MDCK cells being exposed to oxalate ions [91]. However, it has to be noted that some cells did not respond to oxalate injury. This may be due to the fact that changes in gene expression could protect from apoptosis and then inhibit from lithiasis [35]. These findings highlight the need for future studies clarifying novel biochemical targets of kidney stone formation and the utility of p38 MAPK inhibitors in preventing stone formation.

### 5.7. Genetic Basis of Kidney Stone Formation

Environmental factors interacting with underlying genetic factors cause rare stone disease [92]. The production of promoters and inhibitors of crystallization depends on proper functioning of the renal epithelial cells. Cellular dysfunction affects the supersaturation of urinary excretion by influencing ions such as calcium, oxalate, and citrate [93]. Some genetic defects which lead to stone formation are shown in Table 3.

### 5.8. Randall's Plaques

Randall's plaques appear to be the precursor's origin of urinary stone development although it is unclear whether it involves in all stone types or not [62]. Moreover, the pathogenesis of Randall's plaque itself is not clearly known [94]. The majority of CaOx stones are found to be attached with renal papillae at the sites of Randall's plaque [26]. It is located at the interstitial basement membrane in loop of Henle [95, 96]. Calcium phosphate (apatite), and purine crystal compositions were identified in plaques, whereas apatite is dominant [97]. Initially, calcium phosphate crystals and organic matrix are deposited along the basement membranes of the thin loops of Henle and extend further into the interstitial space to the urothelium, constituting the so-called Randall plaques. Evidence suggests that a primary interstitial apatite crystal formation secondarily leads to CaOx stone formation [13]. In supersaturated urine, crystals adhere to the urothelium which may enhance subsequent stone growth [98].

Due to renal cell injury, plaque is exposed to supersaturated urine. Renal epithelial cell damage (degradation) products promote heterogeneous nucleation and promotes crystal adherence in renal cells. Randall plaque calcification is triggered by oxidative stress. Cells may express molecules at distal and collecting tubules which act as crystal binding sites such as phosphatidylserine, CD44, osteopontin, and hyaluronan [27, 99]. Renal epithelial cells of the loop of Henle or collecting ducts produce membrane vesicles at the basal side which leads to plague formation [77]. Thus, apatite crystal deposits have been proposed to act as nidus for CaOx stone formation by attachment on further matrix molecules [13, 77]. However, the driving forces in plaque formation and the involved matrix molecules remain elusive.

Kidney stones are either attached to the renal papillae or found freely [100]. According to the fixed particle pathway, the beginning of calcium phosphate (CaP) deposition in the interstitium establishes a nucleus for CaOx formation. CaP formed in the basement membrane of the loops of Henle, the inner medullary collecting ducts, and ducts of Bellini serves as an attachment site for stone development. Idiopathic stone formers develop CaOx attached to fixed sites of interstitial plaque [26]. Stones of the distal tubular acidosis attach to plugs protruding from dilated ducts of Bellini, whereas cystinuria stones do not attach to the renal plagues (found freely) [26]. CaP, uric acid, or cystine crystals formed in the renal tubules plug at the terminal collecting ducts. When mineralization reaches the renal papillary surface, plaques rupture exposing CaP crystals to the pelvic urine. Then, urinary macromolecules deposit over the exposed CaP crystals and promote CaOx deposition on CaP [4].

### 5.9. Kidney Stone Inhibitors and Promoters

Inhibitors are substances which decrease the initiation of supersaturation, nucleation, crystal growth, rate of aggregation, or any other processes required to stone formation [34]. Normally, urine contains chemicals that prevent crystal formation. Inhibitors in urine includes small organic anions such as citrate, small inorganic anions such as pyrophosphates, multivalent metallic cations such as magnesium, or macromolecules such as osteopontin, glycosaminoglycans, glycoproteins, urinary prothrombin fragment-1, and Tamm–Horsfall proteins [41, 62]. These inhibitors do not seem to work equally for everyone; therefore, some people form stones. But, if crystals formed remain tiny, usually it travels through the urinary tract and passes out from the body with urine splash without being noticed. Inhibitors may act either directly by interacting with crystal or indirectly by influencing the urinary environment [42]. When inhibitory compounds adsorb onto the surface of the crystal, it inhibits nucleation, crystal growth, aggregation, or crystal-cell adherence.

In contrast, promoters are substances which facilitate stone formation by various mechanisms [62]. Some of the promoters include cell membrane lipids (phospholipids, cholesterol, and glycolipids) [42], calcitriol hormone enhancement via parathyroid hormone stimulation [101], oxalate, calcium, sodium, cystine, and low urine volume [34]. Among recurrent stone formers, urinary oxalate excretion was found to be higher, whereas citrate excretion was lower [102]. Studies indicated that oxalate can increase chloride, sodium, and water reabsorption in the proximal tubule and activate multiple signaling pathways in renal epithelial cells [103]. In general, an imbalance between urinary stone inhibitors and promoters has been suggested to be the cause for stone formation [34]. A list of substances generally considered to inhibit or promote stone formation process is shown in Table 1.

## 6. Preventive Options for Urolithiasis

Effective kidney stone prevention depends upon addressing the cause of stone formation. Generally, to prevent the first episodes of kidney stone formation or its secondary episodes, proper management of diet and the use of medications is required. Primary prevention of kidney stone disease via dietary intervention is low-cost public health initiative with massive societal implications. Thus, nutritional management is the best preventive strategy against urolithiasis [104].

Regardless of the underlying etiology and drug treatment of the stone disease, patients should be instructed to increase their water intake in order to maintain a urine output of at least 2 liter per day [49]. A simple and most important lifestyle change to prevent stone disease is to drink more water/liquids. Enough fluid intake reduces urinary saturation and dilutes promoters of CaOx crystallization. Dietary recommendations should be adjusted based on individual metabolic abnormalities. For absorptive hyperoxaluria, low oxalate diet and increased dietary calcium intake are recommended [61].

A high sodium intake boosts stone risk by reducing renal tubular calcium reabsorption and increasing urinary calcium [105]. Restriction of animal proteins is also encouraged since animal proteins provide an increased acid load because of its high content of sulfur-containing amino acids. Thus, high protein intake reduces urine pH and the level of citrate and enhances urinary calcium excretion via bone reabsorption. Therefore, if you have very acidic urine, you may need to eat less meat, fish, and poultry and avoid food with vitamin D [106]. Instead, an increase intake of fruits and vegetables rich in potassium is recommended [49].

People who form calcium stones used to be told to avoid dairy products and other foods with high calcium content. However, persons with a tendency of kidney stone formation should not be advised to restrict calcium intake unless it has been known that he/she has an excessive use of calcium [107]. A reduced intake of calcium leads to an increased intestinal absorption of oxalate, which itself may account for an increased risk of stone formation. Calcium supplements may reduce oxalate absorption because the calcium binds dietary oxalate in the gut lumen. However, the benefit of taking calcium pills is controversial. Vitamin C has been implicated in stone formation because of in vivo conversion of ascorbic acid to oxalate. Therefore, a limitation of vitamin C supplementation is recommended [105].

For prevention of calcium oxalate, cystine, and uric acid stones, urine should be alkalinized by eating a diet high in fruits and vegetables, taking supplemental or prescription citrate, or drinking alkaline mineral waters. For uric acid stone formers, gout needs to be controlled, and for cystine stone formers, sodium and protein intakes need to restricted. For prevention of calcium phosphate and struvite stones, urine should be acidified. For struvite stones, acidifying the urine is the single most important step [108]. Patients must receive careful follow-up to be sure that the infection has cleared. However, the current treatment modalities are not efficient to prevent urolithiasis, and further research is required.

## 7. Conclusion

Despite considerable improvements in the development of new therapies for the management of urinary stones, the incidence of urolithiasis is increasing worldwide. Many aspects of renal stone formation remain unclear. However, it is certain that renal cell injury, crystal retention, cell apoptosis, Randall's plaque, and associated stone inhibitors or promoters play important roles for kidney stone formation. These seem to be critical targets that lead to developing a novel strategy to prevent kidney stone disease and drugs against kidney stones. In addition, the identification of novel treatment targets on the basis of molecular and cellular alterations in relation to stone formation will help develop better drugs. Moreover, better understanding of the mechanisms of urolithiasis associated with stone inhibitors or promoters will be critical for stone-removing medications. Furthermore, understanding the underlying pathophysiology, pathogenesis, and genetic basis of kidney stone formation will hopefully lead to discover novel drugs and strategies to manage urolithiasis in the near future.

## Figures and Tables

**Table 1:** Urinary stone matrix protein modulators of crystallization in nephrolithiasis [34, 41].

Serial Number	Name of protein	Role in crystallization			
		Nucleation	Growth	Aggregation	Cell adherence
1	Nephrocalcin (NC)	I	I	I	—
2	Tamm–Horsfall protein (THP)	P	—	I/P	—
3	Osteopontin/uropontin (OPN)	I	I	I	I/P
4	Albumin	P	—	I	—
5	Urinary prothrombin fragment-1 (UPTF1)	I	I	I	—
6	Alpha-1-microglobulin	—	—	I	—
7	S100A	—	I	I	—
8	Inter-alpha-inhibitor	I	I	I	I
9	Bikunin	I	I	I	I
10	Renal lithostathine	—	I	—	—
11	Alpha defensin	—	P	P	—
12	Human phosphatecytidylyl transferase 1, choline, beta	—	I	—	—
13	Myeloperoxidase	—	P	P	—
14	Nucleolin	—	—	—	P
15	Histone-lysine N methyltransferase	—	I	I	—
16	Inward rectifier K channel	—	I	I	—
17	Protein Wnt-2	—	I	I	—
18	Alpha-2HS glycoprotein	P	I	—	—
19	Crystal adhesion inhibitor (CAI)	—	—	—	I
20	Hyaluronic acid (HA)	—	—	—	P
21	Chondroitin sulphate	—	I	I	—
22	Heparin sulphate (HS)	—	I	—	—
23	Human urinary trefoil factor 1(THF1)	—	I	—	—
24	Monocyte chemoattractant protein-1 (MCP 1)	—	—	—	P
25	Annexin II	—	—	—	P
26	CD44	—	—	—	P
27	Matrix Gla protein (MGP)	—	I	—	I
28	Histone H1B	—	P	—	—
29	Fibronectin	—	—	I	I
30	Collagen	P	—	—	—
31	Glycosaminoglycans	I	I	I	I
32	Citrate	—	I	—	—
33	Pyrophosphate	—	I	—	—
34	Magnesium	—	I	—	—

I: inhibitor; P: promoter; “—”: no effect.

**Table 2:** Risk factors associated with kidney stone formations.

Number	Risk factors	References
1	*Lifestyle habits and dietary/nutritional factors*: such as excessive intake of animal proteins and salt and deficiencies of chelating agents like citrate, fiber, and alkali foods	[9, 13, 19, 45]
2	*Metabolic disorders*: such as hypercalciuria, hypocitraturia, hyperoxaluria, hyperuricosuria, and history of gout (defective metabolism of uric acid)	[38, 46–48]
3	*Hypercalcemic disorders*: primary hyperparathyroidism and other disturbances of calcium metabolism	[49]
4	*Urine composition*: excessive excretion of promoters of urinary crystallization and reduced excretion of inhibitors (urine deficient in inhibitory substances)	[1, 45, 49]
5	*Low urine volume*: inadequate water intake (dehydration and supersaturated urine)	[45, 49, 50]
6	*Recurrent urinary tract infections*: abnormalities of urinary pH and alkalinization of urine by bacterial urease (such as *Proteus mirabilis*)	[38, 49]
7	*Genetic predisposition/inherited disorders*: family history of stones (*genetic* susceptibility); genetic monogenic diseases (single abnormal gene disorders on the autosomes); renal tubular acidosis	[1, 9, 48, 49, 51]
8	*Anatomical abnormalities*: factors such as defects in medullary sponge kidney, ureteropelvic junction stenosis, pyeloureteral duplication, polycystic renal disease, and horseshoe kidney	[1, 48, 49, 52]
9	*Hypertension*	[46]
10	*Obesity*	[46–48]
11	*Climate change* (global warming), occupation, geographic conditions, and seasonal variations (higher in summer than winter)	[1, 49]
12	*Inflammatory bowel disease* and other intestinal malabsorption or associated disease states	[9, 49]
13	Absence of intestinal *oxalate-degrading bacteria*	[53, 54]
14	*Lithogenic drugs*: such as indinavir (Crixivan), a protease inhibitor, sulfonamides (sulfadiazine), uricosuric agents, which have low solubility andpromotes the formation of calculi, and ceftriaxone (high dose on long terms)	[28, 38, 49, 50]

**Table 3:** Gene involved in hypercalciuria, gene products, and renal phenotype [93].

Gene	Gene product/function	Renal phenotype
VDR	Vitamin D receptor	Decreased calcium reabsorption leading to hypercalciuria and nephrocalcinosis
CLCNS	Cl/H antiporter	Inactivating mutation causes hypercalciuria, hyperphosphaturia, low molecular weight proteinuria, nephrocalcinosis, stone
CASR	Calcium sensing receptor	Gain of function mutation produces hypercalciuria, nephrocalcinosis, stone
CLDN16	Tight junction protein	Hypercalciuria, magnesium wasting, nephrocalcinosis, stone
NPT2a/c	Sodium phosphate cotransporter	Hypercalciuria, hypophosphatemia, phosphate wasting, nephrocalcinosis, stone
TRPV5	Calcium selective transient receptor potential channel	Hypercalciuria, hyperphosphaturia
sAC	Soluble adenylate cyclase/bicarbonate exchanger/	Hypercalciuria, stones
KLOTHO	Aging suppression protein/regulator of calcium homeostasis	Hypercalciuria
